# Development of Molecular Resources for an Intertidal Clam, *Sinonovacula constricta*, Using 454 Transcriptome Sequencing

**DOI:** 10.1371/journal.pone.0067456

**Published:** 2013-07-25

**Authors:** Donghong Niu, Lie Wang, Fanyue Sun, Zhanjiang Liu, Jiale Li

**Affiliations:** 1 Key Laboratory of Freshwater Aquatic Genetic Resources Certificated by Ministry of Agriculture, Shanghai Ocean University, Shanghai, China; 2 Shanghai Engineering Research Center of Aquaculture, Shanghai, China; 3 Department of Fisheries and Allied Aquacultures, Auburn University, Auburn, Alabama, United States of America; University of Texas, United States of America

## Abstract

**Background:**

The razor clam *Sinonovacula constricta* is a benthic intertidal bivalve species with important commercial value. Despite its economic importance, knowledge of its transcriptome is scarce. Next generation sequencing technologies offer rapid and efficient tools for generating large numbers of sequences, which can be used to characterize the transcriptome, to develop effective molecular markers and to identify genes associated with growth, a key breeding trait.

**Results:**

Total RNA was isolated from the mantle, gill, liver, siphon, gonad and muscular foot tissues. High-throughput deep sequencing of *S. constricta* using 454 pyrosequencing technology yielded 859,313 high-quality reads with an average read length of 489 bp. Clustering and assembly of these reads produced 16,323 contigs and 131,346 singletons with average lengths of 1,376 bp and 458 bp, respectively. Based on transcriptome sequencing, 14,615 sequences had significant matches with known genes encoding 147,669 predicted proteins. Subsequently, previously unknown growth-related genes were identified. A total of 13,563 microsatellites (SSRs) and 13,634 high-confidence single nucleotide polymorphism loci (SNPs) were discovered, of which almost half were validated.

**Conclusion:**

De novo sequencing of the razor clam *S. constricta* transcriptome on the 454 GS FLX platform generated a large number of ESTs. Candidate growth factors and a large number of SSRs and SNPs were identified. These results will impact genetic studies of *S. constricta*.

## Introduction

The razor clam *Sinonovacula constricta*, a member of the phylum Mollusca (Bivalvia), lives in the lower-to-mid intertidal zones along the coast of the West Pacific Ocean. It is an important benthic shellfish with high commercial value, and it is one of the four major clam species produced by aquaculture in China. In 2009, the cultured razor clam yield was approximately 700,000 tons, which accounted for 30% of the mudflat shellfish production in China [1]. However, the *S. constricta* brood stocks, consisting of mature individuals that improve seed quality and number, are wild populations that have not been genetically selected for beneficial phenotypes.

With aquaculture species, growth-related traits have been the main focus of genetic breeding programs because profits increase when the culture time to maturity is shortened. The identification of QTLs and genes associated with growth traits can enhance selection programs, as demonstrated with aquaculture species [2]. To date, there have been few studies on QTLs affecting growth-related traits in shellfish. QTL analyses in several aquaculture species such, as salmonids [3], tilapia [4], sea bass [5], [6], oyster [7], clam [8], and scallop [9], [10], have demonstrated the feasibility of genetic analysis using molecular markers. High-density genetic linkage maps are required for QTL analysis. Construction of a fine-tuned linkage map requires a large number of molecular markers, especially sequence-tagged microsatellite and SNP markers with co-dominant inheritance [7]. In addition to genetic approaches, molecular biology approaches can identify candidate genes involved in performance traits [11]. The association between candidate gene polymorphisms and traits has been evaluated with genetic markers [2]. By candidate gene screens, some SNPs have been associated with economically valuable traits in fish species, including Atlantic cod [12], gilthead sea bream [13], largemouth bass [14], and Asian sea bass [15]. However, only a few functional genes are associated with growth traits in bivalves. For example, polymorphisms in the amylase gene in *Crassostrea gigas* [16], [17], the myostatin gene in *Chlamys farreri* [18] and *Argopecten irradians* [19], and the insulin-related protein gene in *C. gigas* [20] have been associated with enhanced growth.

The lack of genomic resources coupled with the poor understanding of the molecular and biochemical processes of growth have hindered advances in aquaculture productivity. Sequencing and analysis of expressed sequence tags (ESTs) has been a primary tool for the discovery of novel genes, especially in non-model species. Next generation sequencing (NGS) allows rapid, cost-effective high-throughput sequencing [21]. Understanding gene functions and their effects on phenotypes will be fundamental to future breeding programs [22]. To this end, transcriptome sequencing has been conducted in several shellfish species, including *Meretrix meretrix* [23], *Patinopecten yessoensis* [24], *Ruditapes philippinarum* [25], *Crassostrea angulata* [26], and *C. gigas* [27].

Because the razor clam (*S. constricta*) is an important aquaculture species, a genetic improvement program was initiated in 2006. Consequently, molecular markers have been developed [28], and analyses of population genetics, structure and diversity [29], [30] and functional gene expression [31] have been completed. A small collection of ESTs was generated using traditional Sanger sequencing [32], but large-scale EST resources are not available for the razor clam. We used 454 GS FLX sequencing to generate over 800 million bases of high-quality DNA from the razor clam. Here, we report the generation, assembly and annotation of the transcriptome, and the mining of molecular markers, such as SSRs and SNPs from ESTs.

## Results and Discussion

### Sequencing and Assembly

**Figure 1:**
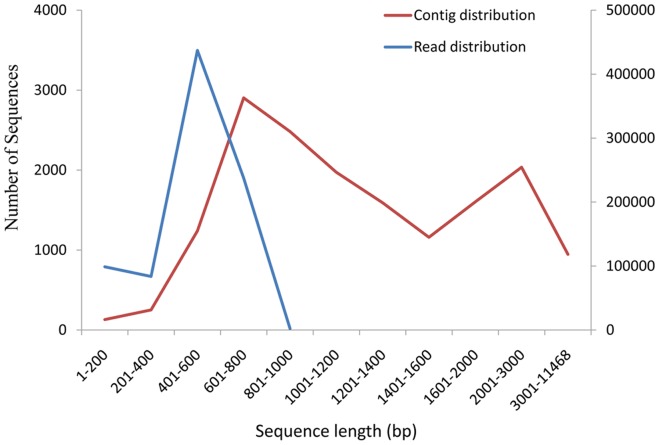
Length distribution of total reads and contigs from the *S. constricta* transcriptome.

This study is the first to comprehensively describe the *S. constricta* transcriptome. This Transcriptome Shotgun Assembly project has been deposited at DDBJ/EMBL/GenBank under the accession GALB00000000. The version described in this paper is the first version, GALB01000000. In total, 859,313 reads with an average length of 489 bp were obtained from a single run on the Roche 454 GS FLX sequencing platform (**Table 1**). Most reads (65.5%) were 441–680 bp (**Figure 1**). After eliminating low quality reads (those trimmed from both ends due to low (<20) quality scores), sequence assembly yielded a non-redundant set of 147,669 ESTs, containing 16,323 contigs and 131,346 singletons with average lengths of 1,376 bp and 458 bp, respectively. Most contigs (57.1%) were larger than 1 kb (**Figure 1**), with the longest contig containing 11,468 bp. These transcriptome sequences contained 3845 ESTs that matched to cDNAs identified in a previous study (5296 ESTs) [32].

**Table 1:** Comparison of experiment conditions and analysis results among *Sinonovacula constricta*, *Ruditapes philippinarum, Chamelea gallina* and *Meretrix meretrix*.

Dataset name	*Sinonovacula constricta*	*Chamelea gallina*	*Meretrix meretrix*	*Ruditapes philippinarum*
Sequencing method	454	454	454	454
Individuals tested	6	4	-	20
Tissues tested	mixture	muscle	mixture	mixture
Development stage	adult	adult	larval	adult
Total number of reads	859,313	298,369	751,970	457,717
Number of contigs	16,323	41,630	35,205	32,606
Average length of contigs	1,376	335	679	546
Number of singletons	144,252	96,244	89,532	266,093
Number of annotated genes	14,615 (E≤1E−5)	18,196 (E≤1E−3)	16,070 (E≤1E−10)	9,747 (E≤1E−3)
Identified SNP	13,634	20,377	-	-
Identified SSR	13,563	111	-	-
Identified genes	Growth factor	Pathogenic sequences	Related to development and growth	Related to environmental monitoring
Reference	This study	Coppe, 2012. [43]	Huan, 2012. [23]	Milan, 2011 [44]

### Sequence annotation

**Figure 2:**
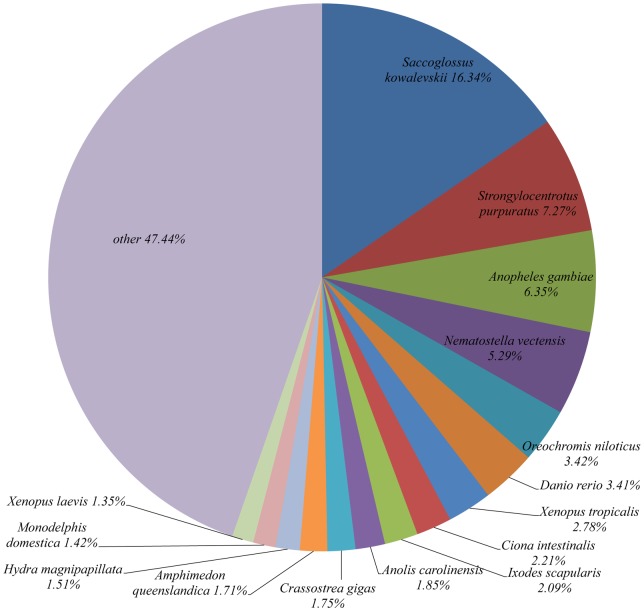
Species matched to the annotated sequences of *S. constricta* by BLASTx.

**Figure 3:**
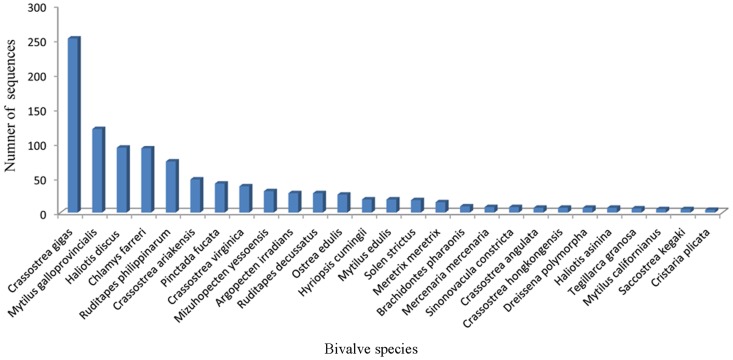
Bivalve species matched to the annotated sequences of *S. constricta*.

The 16,323 contigs and 131,346 singletons were used as queries to search against the non-redundant protein database on NCBI using. BLASTx (E-value≤1e-5). Of the 147,669 sequences, 14,615 (9.9%) had significant matches to known genes, with 3,066 significant hits from the contigs and 11,549 significant hits from singletons. The relatively low rate of putative identifications via BLAST analysis is not unusual with invertebrates [24], [26], [33]. The low annotation rate could be attributed to insufficient information in the public database from non-model species, especially from bivalves, which have a distant phylogenetic relationship with well-studied species such as mammals. This assessment is consistent with the fact that most matches (51.0%) were from invertebrate species or non-mammalian vertebrates, including *Saccoglossus kowalevskii*, *Strongylocentrotus purpuratus*, *Anopheles gambiae*, *Nematostella vectensis*, *Oreochromis niloticus*, *Danio rerio*, *Xenopus tropicalis*, *Ciona intestinalis, Ixodes scapularis*, and *Anolis carolinensis* (**Figure 2**). A total of 1,019 sequences (6.9%) matched to 27 bivalve species; the top five species were *C. gigas* (17.6%), *Mytilus galloprovincialis* (8.4%), *Haliotis discus* (6.5%), *Chlamys farreri* (6.5%), and *Ruditapes philippinarum* (5.1%) (**Figure 3**). Due to the limited genetic resources, fewer sequences matched to bivalve species. Only eight annotated sequences matched to *S. constricta*, and these sequences included coding regions for tropomysin, ferritin, ATP9, NADH2, and cyb.

**Figure 4:**
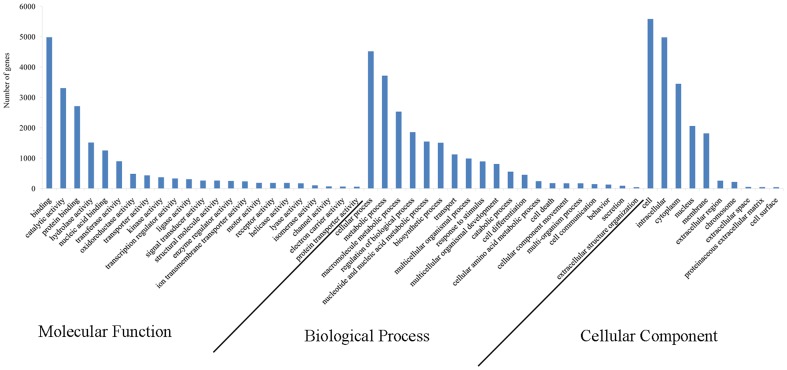
Gene ontology (GO) terms for the transcriptome sequences of *S. constricta*.

Gene Ontology (GO) terms were assigned to the deduce protein sequences based on their sequence similarities to known proteins in the Swiss-Prot and TrEMBL databases. A total of 6,663 deduce protein sequences were assigned 4,724 GO terms, which were distributed under the three main categories of Molecular Function, Biological Process and Cellular Components. A detailed distribution of genes in the main ontology is illustrated in **Figure 4**. Within the Molecular Function category, genes encoding binding proteins and proteins related to catalytic activity were the most enriched. Proteins related to metabolic processes and cellular processes were enriched in the Biological Process category. In the Cellular Components category, the cell and cell part were the most highly represented categories. The composition and distribution of assigned GO terms from other mollusks, such as *Crassostrea angulata* [26], *Patinopecten yessoensis* [24] and *Meretrix meretrix* [23], were very similar, indicating conserved genes or metabolic pathways. Alternatively, this result may indicate that genes encoding these functions are more conserved between different organisms and thus easier to annotate [34]. Moreover, the high expression of hydrolytic enzymes and metabolic genes may favor metabolic activities that promote fast growth [23], [35]. Functional annotation is a prerequisite for understanding transcriptome data (especially of non-model systems) [35], as it allows for the analysis of unknown sequences and aids in the investigation of specific pathways, cellular structures and protein functions [26]. The results presented here will help identify unknown growth and reproduction genes.

### Identification of growth-related genes

Growth factor-related genes promote cell division and maturation as well as tissue growth and remodeling. The insulin-like growth factor (IGF) system is composed of two ligands (IGF-1, IGF-2), two receptors (IGF-1R, IGF-2R) and six IGF-binding proteins (IGFBPs) [36]. In qPCR experiments of *Haliotis midae* in vivo and in vitro, genes in the insulin signaling pathway were up-regulated, suggesting that insulin may be involved in enhanced growth [37]. MSTN, also known as growth and differentiation factor 8 (GDF8), is a negative regulator of vertebrate muscle growth. MSTN SNPs are significantly associated with growth traits in the commercial scallop [18], bighead carp (*Aristichthys nobilis*) [38], and spotted halibut (*Verasper variegatus*) [39]. In this study, we identified a number of growth-related genes, including growth factors, growth factor receptors, and growth factor-binding proteins (**Table 2**), which have been rarely reported in bivalves. These gene sequences should be further studied for their association with growth and development.

**Table 2:** Identification of growth factors, growth factor receptors, and growth factor binding proteins by BLASTx.

Gene name	Length (aa)	Hits	E-value
Insulin-related protein	181	1	5E-7
Insulin-like growth factor-binding protein	116–380	5	7E-20∼1E-55
Insulin related protein receptor	66–205	8	2E-6∼4E-37
Fibroblast growth factor receptor	74–507	27	3E-6∼4E-80
Fibroblast growth factor -binding protein	135–139	3	2E-28∼5E-41
Epidermal growth factor-like domains	96–275	56	1E-6∼2E-38
Epidermal growth factor receptor	191–185	2	3E-18∼6E-42
Growth/differentiation factor 8 (MSTN)	142–484	2	4E-09∼7E-56
Transforming growth factor beta regulator	99	1	4E-19
Transforming growth factor-beta			
receptor-assotiation protein	132–229	5	2E-08∼3E-26
transforming growth factor			
beta-binding protein	122	1	3E-09
Hepatocyte growth factor receptor	82–237	20	1E-11∼7E-60
Opioid growth factor receptor	136–185	5	2E-08∼3E-31

### Discovery and validation of molecular makers

**Figure 5:**
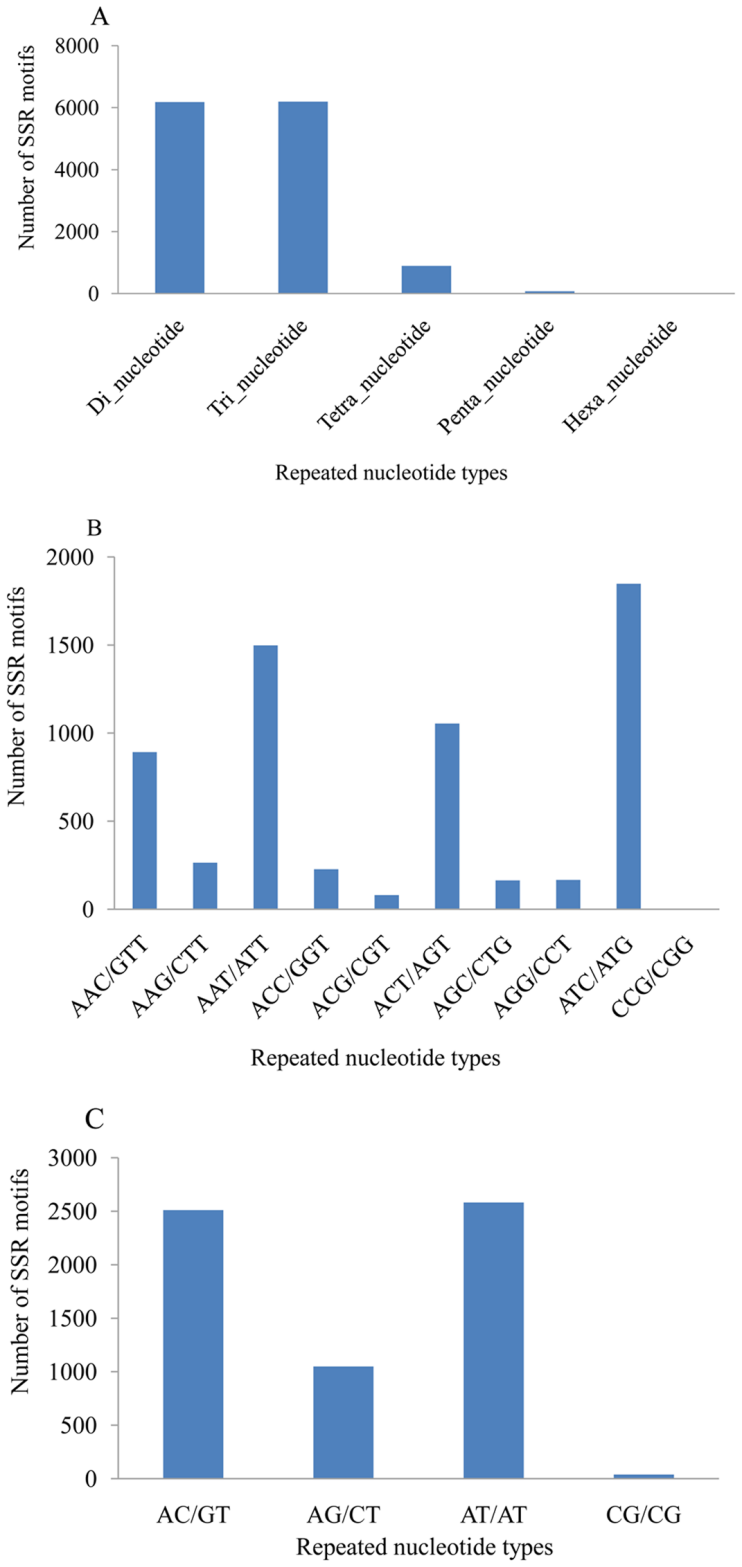
Distribution of simple sequence repeats (SSR) and other nucleotide repeats in the transcriptome. (A) Distribution of five nucleotide repeat types (di-, tri-, tetra-, penta-, and hexa-nucleotide repeats). (B) Distribution of tri-nucleotide repeats. (C) Distribution of di-nucleotide repeats. SSRs had at least six di-nucleotide repeats and five other repeats (tri-, tetra-, penta-, and hexa-nucleotide repeats).

The development of microsatellite markers is time-consuming and expensive, as it requires preparation of genomic libraries, hybridization to detect positive clones, plasmid isolation and sequencing [40]. Next-generation sequencing provides an efficient and cost-effective way to identify microsatellites [41]. Using SciRoKo v3.4 [42], we identified 13,563 microsatellites, which consisted of 1,583 and 11,980 in the assembled contigs and singletons, respectively. Most microsatellites were di-nucleotide (46.3%) and tri-nucleotide (46.4%) repeats (**Figure 5A**). (AAT/ATT)_n_ and (ATC/ATG)_n_ were the predominant tri-nucleotide repeat motifs, with frequencies of 11.2% and 13.8%, respectively (**Figure 5B**). (AC/GT)_n_ and (AT/TA)_n_ were the predominant di-nucleotide repeat motifs types, with frequencies of 18.8% and 19.3%, respectively (**Figure 5C**). These microsatellites can be used for future population genetics and mapping studies.

To evaluate these identified microsatellites, we designed 55 pairs of primers with 5′ fluorescent dye (FAM) labels and screened 24 individual *S. constricta*. No product and/or non-specific bands occurred for 19 primer pairs, and 10 primers produced monomorphic PCR products. Polymorphisms were detected with the remaining 26 primer sets. The number of effective alleles (*Ar*) per locus varied from three to 20, and the values of observed heterozygosity (*Ho*) and expected heterozygosity (*He*) ranged from 0.250 to 0.917 and from 0.620 to 0.950, respectively (**Table S1**). These results suggested that almost half of the identified microsatellites could be validated and used for various genetic studies.

**Figure 6:**
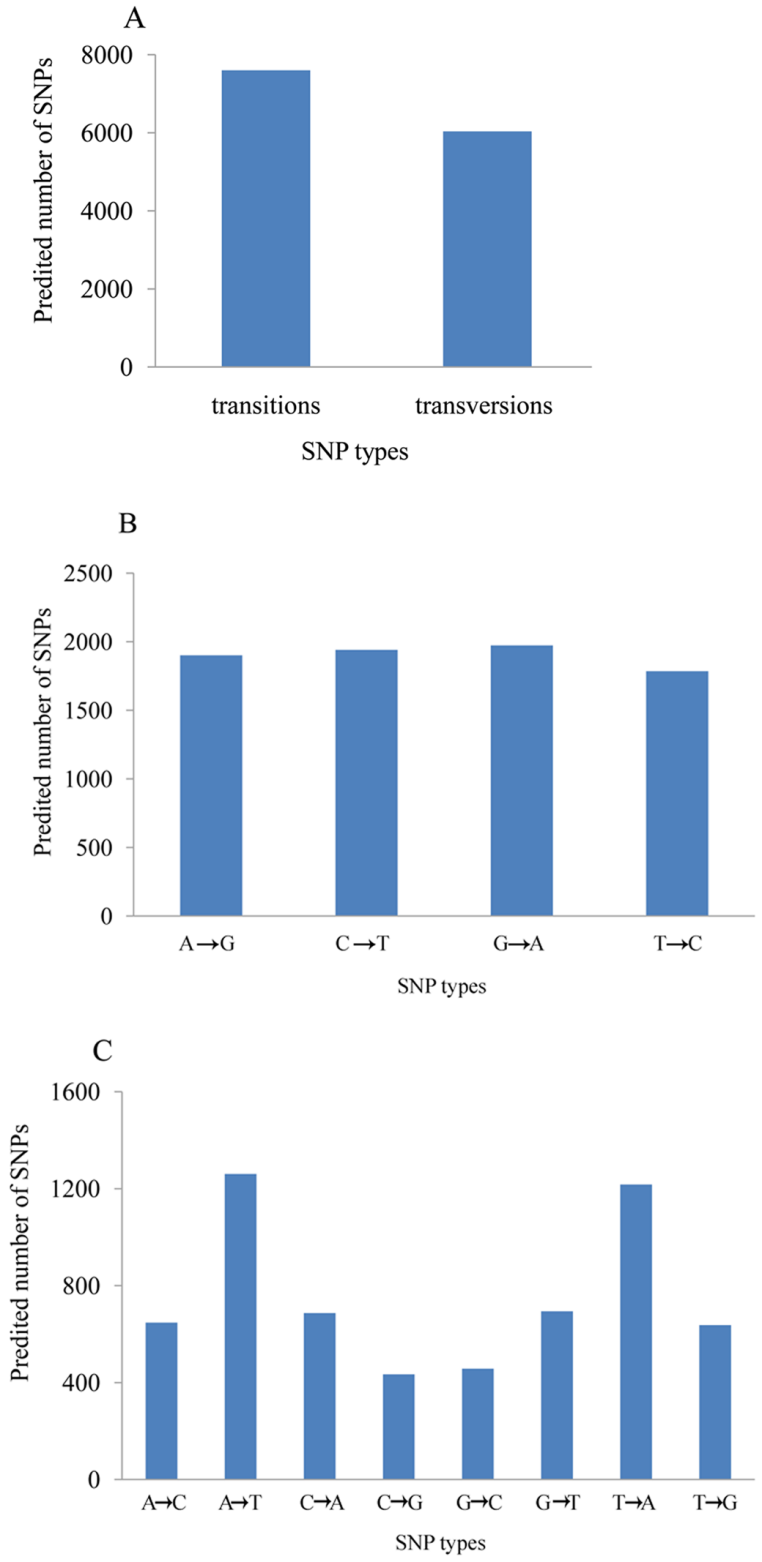
Distribution of predicted single nucleotide polymorphisms (SNPs) in the transcriptome. (A) Distribution of total transitions and transversions. (B) Distribution of each transition. (C) Distribution of each transversion.

Of the 13,634 identified SNPs, 7,600 loci were transitions and 6,034 loci were transversions (**Figure 6**). To validate potential SNPs, a subset of 26 ESTs containing 47 SNPs was selected randomly. These SNP loci were amplified from the DNA of six *S. constricta* individuals. PCR products were Sanger sequenced with forward and reverse primers on an ABI3730 platform (Applied Biosystems). Of the 47 SNP loci predicted in the amplified sequences, 40 (85.1%) were validated by apparent polymorphisms (**Table S2**).

### Comparison of transcriptomes from four Veneridae bivalves

**Figure 7:**
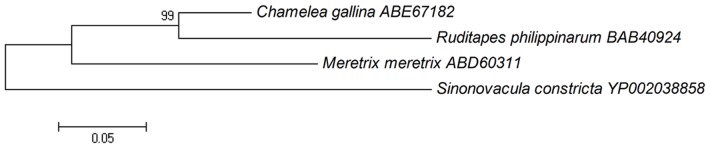
NJ phylogenetic tree based on Mt-COI protein sequences from four Veneridae species (*S. constricta*, *C. gallina*, *M. meretrix*, and *R. philippinarum*).

**Figure 8:**
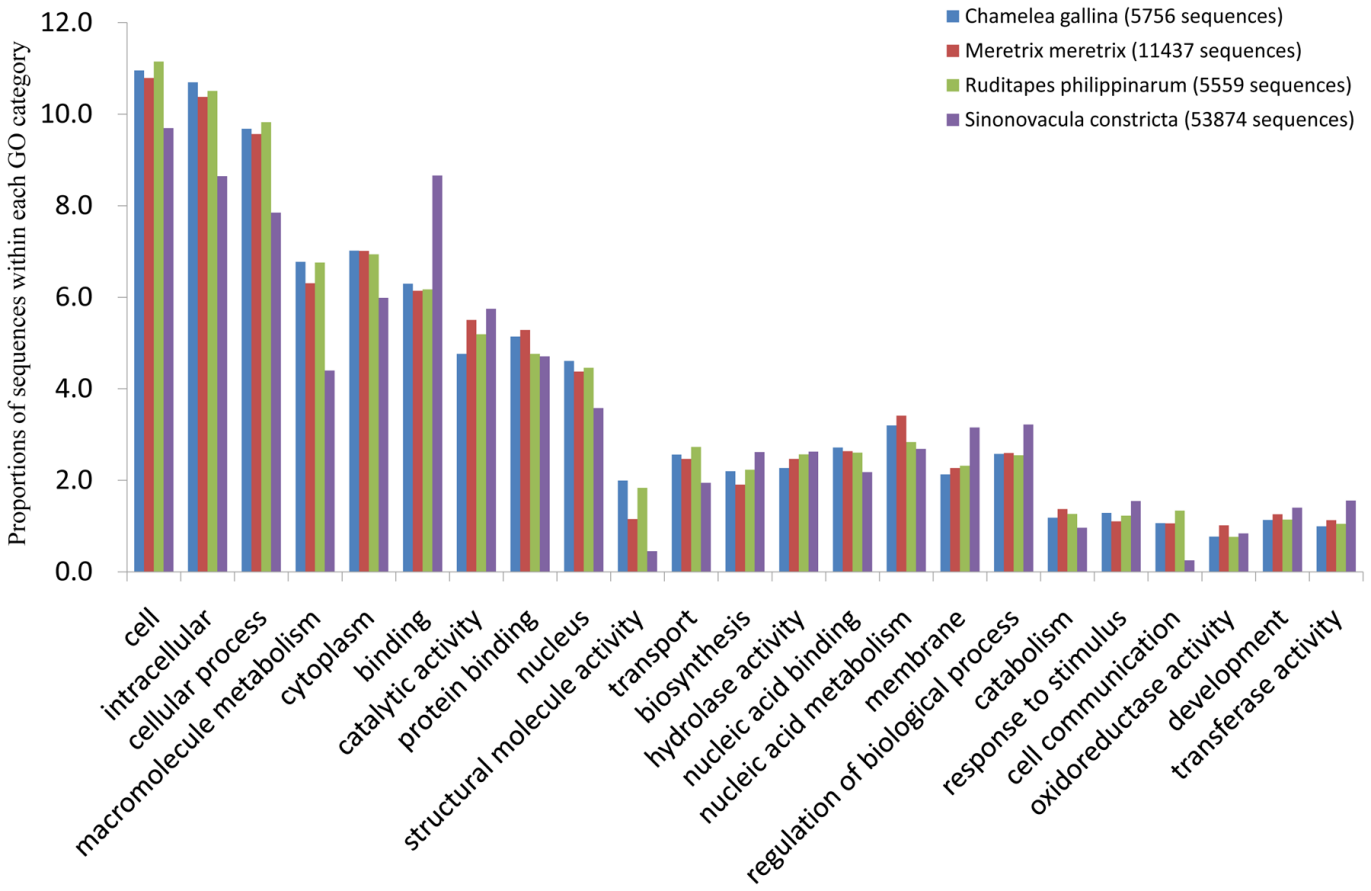
Comparison of gene ontology (GO) in transcriptomes from *C. gallina*, *M. meretrix, R. philippinarum* and *S. constricta*.

The transcriptomes of three other Veneridae family bivalve species, *C. gallina* [43], *M. meretrix* [23], and *R. philippinarum* [44], previously sequenced on the 454 platform were downloaded from NCBI. These datasets included 165,283 *C. gallina* reads, 35,004 *M. meretrix* contigs and 457,667 *R. philippinarum* reads. By comparing these datasets with our experimental conditions and analysis (**Table 1**), we found that our *S. constricta* transcriptome assembly had longer contigs (average length of 1,367 bp). We compared the transcriptomes of these species and *S. constricta* using BLASTn (E≤1e-10), with the following results: 920 *S. constricta* contigs matched 4,606 of *C. gallina* reads; 1,468 *S. constricta* contigs matched 2,091 of *M. meretri x* contigs; and 983 *S. constricta* contigs matched 18,505 *R. Philippinarum* reads. Using BLASTn with the transcriptomes of *C. gallin*, *M. meretrix* and *R. philippinarum* and singletons from *S. constricta*, the matched values were 3576/3468, 4124/1313 and 3814/16368, respectively. Based on the matched unigenes between sets of bivalves (*M. meretrix* and *S. constricta*: 1447 genes; *C. gallina* and *S. constricta*: 728 genes; *R. philippinarum* and *S. constricta*: 705 genes), *M. meretrix* and *S. constricta* appear most closely related. To further examine genetic relationships, we constructed an NJ phylogenetic tree based on mitochondrial COI protein sequences, as Mt-COI sequences are used for barcoding and verifying species [45], [46] (**Figure 7**). *C. gallina* and *R. philippinarum* clustered together, and the next closest branch contained *M. meretrix*. By comparison of GO analyses for each bivalve species, we found that the matched genes were primarily classified as cell and intracellular genes in the Cellular Component category, cellular process and macromolecular metabolism genes in the Biological Process category, and binding, catalytic activity and protein binding genes in the Molecular Function category (**Figure 8**). The major functions determined by GO were similar in each bivalve transcriptome.

## Materials and Methods

### Ethics Statement

Clams were handled in accordance with the guidelines on the care and use of animals for scientific purposes set by the Institutional Animal Care and Use Committee (IACUC) of Shanghai Ocean University, Shanghai, China.

### Tissue material and RNA extraction

Six adult individuals of *S. constricta* were obtained from Ninghai City, Zhejiang Province, China in 2011. Mantle, gill, liver, siphon, gonad and foot tissues were dissected, immediately frozen in liquid nitrogen and stored at −80°C.

Total RNA was extracted from the tissues with TRIzol Reagent (Invitrogen, USA) according to the manufacturer's instructions. The concentration of total RNA was determined by NanoDrop (Thermo Scientific, USA), and the RNA integrity value (RIN) was checked with a RNA 6000 Pico LabChip on an Agilent 2100 Bioanalyzer (Agilent, USA).

### Library construction and 454 pyrosequencing

cDNA libraries were prepared at the Chinese National Human Genome Center in Shanghai. Double-stranded cDNA was synthesized following the manufacturer's protocol [47]. First-strand cDNA synthesis included a GsuI-oligodT primer, 10 µg of mRNA, and 1000 units of Superscript II reverse transcriptase (Invitrogen). After incubation at 42°C for 1 hr, the 5′mRNA CAP structure was oxidized by NaIO4 (Sigma) and ligated to biotin hydrazide, which was used bind complete mRNA/cDNA to Dynal M280 beads (Invitrogen). After second-strand cDNA synthesis, the polyA tail and 5′ adaptor were removed by GsuI digestion. cDNA size fractionation was performed with a cDNA size fractionation column (Agencourt). Prepared cDNAs were modified into single-stranded template DNA (sstDNA) libraries with a GS DNA Library Preparation kit (Roche Applied Science). sstDNA libraries were clonally amplified in a bead-immobilized form with a GS emPCR kit (Roche Applied Science). After the bead enrichment efficiency was examined, a whole-plate sequencing run was performed with Roche 454 GS FLX Titanium chemistry (Roche Diagnostics, Indianapolis, IN, USA)

### Sequence assembly and annotation

A total of 859,313 sequence reads were produced by 454 pyrosequencing. Reads less than 50 bp and low-quality reads were filtered out, and the remaining 667,713 (75%) high-quality sequence reads were assembled with Newbler 2.7 software with the “cDNA assembly” and “extend low depth overlaps” parameters and all other parameters set to their default values. Functional annotation was performed by BLASTx searches against the non-redundant (nr) protein database in GenBank with an E-value cutoff of E≤1e-5. Newbler 2.7 was used to create a hierarchical assembly composed of contigs, isotigs, and isogroups. Contigs are stretches of assembled reads that are free of branching conflicts. An isotig represents a particular continuous path through a set of contigs. An isogroup is the set of isotigs arising from the same set of contigs. To avoid redundant annotations, we chose the longest ‘isotig’ or ‘contig’ in each ‘isogroup’ to represent the corresponding gene (gene locus). Thus, each ‘isogroup’ was represented by one contig, and all ‘isotigs’ and ‘contigs’ were renamed to a uniform contig number. Gene names were assigned to each sequence based on the best BLAST matches. Gene ontology analysis was conducted using GoPipe [48] (E-value≤1e-5) against the Swiss-Prot database. The BLAST results were utilized by the GoPipe software to annotate the GO terms with built-in statistical options. These results showed that the transcriptome contained gene products involved in biological processes, cellular components and molecular functions of gene products.

### Identification of EST-SSR motifs and EST-SNPs

The sequences were screened for microsatellites using SciRoKo v3.4 software [42]. The criteria for SSRs were sequences having at least six di-nucleotide repeats and five repeats for all other repeats (tri-, tetra-, penta-, and hexa-nucleotide). To detect microsatellite polymorphisms, fifty-five EST-SSR loci with sufficient flanking sequences were selected from the singletons. Primers were designed with PRIMER 3 software to generate 100–300 bp products. Forward primers were 5′ end labeled with a fluorescent dye (FAM). Microsatellite loci were characterized in 24 *S. constricta*i ndividuals from Ninghai City, Zhejiang Province, China. Fragment sizes were determined with the ROX-500 standard using Genescan version 3.1 and Genotyper version 2.1 (Applied Biosystems). The number of effective alleles (*Ar*) and number of observed (*Ho*) and expected (*He*) heterozygosities were estimated with GENALEX 6.0 [49].

SNPs were extracted using VarScan (http://varscan.sourceforge.net) with the default parameter (min. coverage: 8; min reads: 2; min. var. freq.: 0.01; min. avg. qual.:15) only when both alleles were detected in the contigs. Because no reference sequences were available, SNPs were identified as superimposed nucleotide peaks where two or more reads contained polymorphisms at the variant allele. To validate the putative SNPs identified in ESTs, twenty-six EST sequences containing 47 potential SNPs were amplified from six *S. constricta* individuals. PCR products were Sanger sequenced in both directions on the ABI3730 platform (Applied Biosystems). Sequencing chromatograms were visually analyzed with Vector NTI software (Invitrogen), and SNP types were recorded with the genotypes.

## Conclusions

De novo transcriptome sequencing of the razor clam *S. constricta* was conducted on the 454 GS FLX sequencing and generated a large number of ESTs. EST assembly allowed for the identification of 14,615 genes with significant hits to known genes. A large number of microsatellites and SNPs were also identified. Because a small fraction of the microsatellites and SNPs were validated, the remaining putative markers could potentially be validated in the future to provide a rich marker resource for genetic analysis of this important aquaculture species.

## Supporting Information

Table S1 Details of EST-SSR in *S. constricta* including locus name, repeat motif, primer sequence, original size, effective alleles (*Ar*), expected (*He*) and observed (*Ho*) heterozygosities and GenBank accession number.
(DOCX)

Table S2 Details of verifying SNPs including isotig number existing SNPs, primer sequences, SNP position, and SNP types and number.
(DOCX)
