# Supplementary material for: Development of Molecular Resources for an Intertidal Clam, Sinonovacula constricta, Using 454 Transcriptome Sequencing
Source: PLoS One. 2013 Jul 25;8(7):e67456. doi: 10.1371/journal.pone.0067456 (PMC3723811; doi:10.1371/journal.pone.0067456)
Supplement: Table S1 — Details of EST-SSR in S. constricta including locus name, repeat motif, primer sequence, original size, effective alleles (Ar), expected (He) and observed (Ho) heterozygosities and GenBank accession number. (DOCX) [file pone.0067456.s001.docx]

**Supplementary Table 1** Details of EST-SSR in *S. constricta* including locus name, repeat motif, primer sequence, original size, effective alleles (*Ar*), expected (*He*) and observed (*Ho*) heterozygosities and GenBank accession number.

| Locus | Repeat motif | Primer sequence (5’-3’) | Size (bp) | *Ar* | *Ho* | *He* | *GenBank* *No.* |
| --- | --- | --- | --- | --- | --- | --- | --- |
| ScEST-1 | (TG)_15_ | AACAATCCATGTAACCACACCA | 197 | 12 | 0.917 | 0.918 | JX521808 |
|  |  | TTCACATCAAATGGGCACAG |  |  |  |  |  |
| ScEST-2 | (TTA)_23_ | ATTCGCATTGTTGTCGAAGG | 261 | 17 | 0.833 | 0.941 | JX521809 |
|  |  | TCTCGCCTTCTTCTGTCTCC |  |  |  |  |  |
| ScEST-3 | (ATG)_12_ | CACCAACTTGACCAATGACG | 171 | 8 | 0.833 | 0.882 | JX521810 |
|  |  | AAACCAAGTGTGCCCTGAAA |  |  |  |  |  |
| ScEST-7 | (AAT)_43_ | CCAGTGCATATCATACCTGATG | 276 | 8 | 0.375 | 0.880 | JX521811 |
|  |  | CAAGTCTCAAAACAAAGCAG |  |  |  |  |  |
| ScEST-8 | (GTA)_25_ | GGCGCTTTAGGACGTTTTTA | 297 | 10 | 0.417 | 0.898 | JX521812 |
|  |  | CGGCAAAGACTTAAACGTTGTT |  |  |  |  |  |
| ScEST-11 | (ATC)_16_ | TATAGGCGGGTTGAAAAAGC | 171 | 8 | 0.250 | 0.870 | JX521813 |
|  |  | CTCAGCGGCGTTCAATTAG |  |  |  |  |  |
| ScEST-12 | (ATC)_30_ | CATCATTAACCGGTGCCATT | 179 | 3 | 0.792 | 0.620 | JX521814 |
|  |  | TCGTTCTGATGTGGAAAGTCC |  |  |  |  |  |
| ScEST-13 | (TAT)_24_ | CGGCCAATCTAGAGCTCGTT | 262 | 8 | 0.364 | 0.882 | JX521815 |
|  |  | CCATCCCACAAGGGAGATAA |  |  |  |  |  |
| ScEST-15 | (AAT)_11_ | TGATGCAGACCTGTTCACAA | 227 | 14 | 0.333 | 0.928 | JX521816 |
|  |  | TGGGTCCTCATGACACATTG |  |  |  |  |  |
| ScEST-19 | (GAT)_17_ | TGGTGGTGGTAATGATGACG | 139 | 8 | 0.909 | 0.874 | JX521817 |
|  |  | CAATGCGCTTTGGGATCAT |  |  |  |  |  |
| ScEST-20 | (CG)_9_(AC)_7_ | AAGACCGTCCATTGTCCTTG | 194 | 6 | 0.292 | 0.824 | JX521818 |
|  |  | GTGTTGTGAGCGTGCGTGT |  |  |  |  |  |
| ScEST-21 | (CT)_11_ | CTGCTGAGAGCACCAGACAC | 165 | 7 | 0.955 | 0.858 | JX521819 |
|  |  | GAGACGGGGGAGAGAGAGAC |  |  |  |  |  |
| ScEST-23 | (AC)_8_(GCAC)_6_ | ACACCAATGGCTGTTCACTT | 212 | 5 | 0.583 | 0.797 | JX521820 |
|  |  | GCCCTTTATCGTGGTGTTGT |  |  |  |  |  |
| ScEST-24 | (ATC)_26_ | TCCCGATCATCATCATCCTT | 170 | 13 | 0.833 | 0.922 | JX521821 |
|  |  | GAAGACGATCGATACTTGATGC |  |  |  |  |  |
| ScEST-25 | (TCA)_22_ | CAGCAGCCGTATCAGATTCA | 273 | 20 | 0.583 | 0.950 | JX521822 |
|  |  | ACGCCTTGGTTTCTCTGCT |  |  |  |  |  |
| ScEST-30 | (AAC)_8_ | GGCAAAGTTCTGGAATGGAA | 144 | 8 | 0.542 | 0.876 | JX521823 |
|  |  | GACCTGGCACTCGTTATGCT |  |  |  |  |  |
| ScEST-31 | (TG)_14_ | TCTGACAGATATTTGTGTGTGC | 105 | 11 | 0.909 | 0.906 | JX521824 |
|  |  | ATTAATGCGGCTGGCATAAC |  |  |  |  |  |
| ScEST-40 | (ATG)_18_ | TTCGTAGGAACCTGTTCGATTT | 196 | 5 | 0.857 | 0.815 | JX521825 |
|  |  | CACAACCGAGAAGGAGCATT |  |  |  |  |  |
| ScEST-42 | (AAT)_26_ | TGATTAAGACGACGACAACGA | 298 | 12 | 0.636 | 0.915 | JX521827 |
|  |  | CCTTGATTTACTCAACAGCGACT |  |  |  |  |  |
| ScEST-50 | (AGAC)_14_ | AGGTCCTCACCATGCGTAAT | 181 | 5 | 0.409 | 0.779 | JX521828 |
|  |  | TTGAACTCAAGCGGTGACAG |  |  |  |  |  |
| ScEST-52 | (AAT)_14_ | CATTGCCATCTTTGCTGCT  AGCACCTGGCCAAAACATAC | 227 | 5 | 0.750 | 0.791 | JX521829 |
| ScEST-53 | (TG)_7_ | CTCTGCATGCTCTTTCCAGA | 189 | 9 | 0.545 | 0.885 | JX521830 |
|  |  | ATTTGCTTTTGGTGTTGTTT |  |  |  |  |  |
| ScEST-9 | (AAAC)_10_ | GCATTGTTCAAAGGGGTTTT | 176 | 8 | 0.364 | 0.881 | JX521831 |
|  |  | CAAAACGTCGAAGGCTGAAT |  |  |  |  |  |
| ScEST-16 | (TC)_11_ | CAGCCAATGCATAACAAAACC | 214 | 12 | 0.565 | 0.919 | JX521832 |
|  |  | CACGTTGGCAAACTTTTGTG |  |  |  |  |  |
| ScEST-33 | (ATC)_21_ | TGACAGAAACATCACCATCA | 221 | 14 | 0.611 | 0.931 | JX521833 |
|  |  | TCTATGACAGTGCAGAAGATG |  |  |  |  |  |
| ScEST-39 | (ATC)_12_ | ACCTCTGGGCATAGACGACA | 282 | 6 | 0.600 | 0.823 | JX521834 |
|  |  | GGCCTCGTCTTAAGATCACA |  |  |  |  |  |
