# Supplementary material for: Development of Molecular Resources for an Intertidal Clam, Sinonovacula constricta, Using 454 Transcriptome Sequencing
Source: PLoS One. 2013 Jul 25;8(7):e67456. doi: 10.1371/journal.pone.0067456 (PMC3723811; doi:10.1371/journal.pone.0067456)
Supplement: Table S2 — Details of verifying SNPs including isotig number existing SNPs, primer sequences, SNP position, and SNP types and number. (DOCX) [file pone.0067456.s002.docx]

**Supplementary Table 2** Details of verifying SNPs including isotig number existing SNPs, primer sequences, SNP position, and SNP types and number.

| Isotig | Primer (5’-3’) | Position | SNP types/number | | |  |
| --- | --- | --- | --- | --- | --- | --- |
| 20898 | AAGTCAGCGAAAGTGCCAGT | 1035 | AG/5 | AA/1 |  |  |
|  | AAATTGAAGATGCCCGTGAG |  |  |  |  |  |
| 10136 | AAGGCCAACAGGGAAAAGAT | 969 | GA/4 | GG/2 |  |  |
|  | TGGTCTCATGGATACCAGCA | 1071 | CC/6 |  |  |  |
|  |  | 1206 | CT/4 | CC/2 |  |  |
| 17615 | AGTATTTGCGCTCTGGAGGA | 1398 | GG/2 | GA/1 | GT/2 |  |
|  | GCTTCTTCGAGCTCTCTGGA |  |  |  |  |  |
| 11437 | ACCATTGCCAAGTCTGGAAC | 622 | GT/4 | TT/2 |  |  |
|  | CACAGGGGCTTTGTTTTGTT | 640 | CT/1 | CC/4 |  |  |
| 11437 | AACAAAACAAAGCCCCTGTG | 1117 | CT/4 | TT/2 |  |  |
|  | TGGCAAACTGCTCATACAGC |  |  |  |  |  |
| 11437 | AGTACGGATGGTCTGCCAAC | 2071 | AA/6 |  |  |  |
|  | CGCATATGGAGCTCTGATGA | 2074 | AG/4 | GG/2 |  |  |
| 19024 | CCCTCAAAGTCCTCCTCTCC | 967 | CT/3 | TT/1 | CC/2 |  |
|  | TCGCAAAAATTCCCAACTTC | 997 | CT/2 | TT/3 | CC/1 |  |
| 17270 | TGTTTTGGGCTGATTTCTCC | 1096 | GG/6 |  |  |  |
|  | TGAGTGCAAGAACCCATCTG |  |  |  |  |  |
| 17270 | CCAGATTCGTAGGTGGCAAT | 2646 | TG/4 | TT/2 |  |  |
|  | GGAAAGTCCACCCTGACTGA |  |  |  |  |  |
| 27472 | TCCCAAGAAGGGAAATGAAA | 110 | CT/1 | AC/2 | AT/1 | CC/2 |
|  | GGTCAGGGGATACTTGACGA | 137 | CT/3 | CC/3 |  |  |
|  |  | 209 | AG/2 | GG/3 | AA/1 |  |
|  |  | 227 | AG/3 | GG/3 |  |  |
| 6461 | TCGTCACTTTTCAGCAGACG | 652 | AG/1 | AA/2 | GG/3 |  |
|  | ATGTTTGCTCCCACCAAGAC |  |  |  |  |  |
| 23309 | GACCTCATTACGCCTGTGGT | 334 | AG/1 | GG/5 |  |  |
|  | GGCCTTCCCTAAAGGATGAG | 499 | GT/3 | GG/3 |  |  |
| 8253 | GCATCTTCATGGGCAAAACT | 770 | TT/3 | CT/2 | CC/2 |  |
|  | GGCCCATCCATTATGAAGAA | 794 | TC/1 | TT/4 | CC/1 |  |
|  |  | 817 | AT/2 | AA/4 |  |  |
| 12293 | CGCTGATAGACTGCAAGCTG | 2573 | TT/5 | CT/1 |  |  |
|  | CATGGAAGTGGCCTTTCTGT |  |  |  |  |  |
| 26084 | GCGTCATCATCAGCGTAAGA | 236 | TT/2 | CC/2 | CT/2 |  |
|  | GGGTACAGCTAATGCCCGTA |  |  |  |  |  |
| 24753 | TGGGCTCACTGACAAGACTG | 483 | CT/1 | CC/5 |  |  |
|  | ACCTGTGTCGCTTCCTCTGT |  |  |  |  |  |
| 8529 | ACAGCATACCGACCTTCACC | 2255 | GT/6 |  |  |  |
|  | CAAGAGGGAGGCCGTACATA |  |  |  |  |  |
| 19788 | AACAGGCCAAAACGAACATC | 873 | CC/6 |  |  |  |
|  | CTTGTGCCGGTCGTTAATTT |  |  |  |  |  |
| 24093 | GCCAACCGTCTGTCTCTCTC | 478 | CT/2 | CC/4 |  |  |
|  | AGGTGAGCACTTCAGGCTGT | 301 | AG/1 | GG/3 | AA/2 |  |
|  |  | 208 | GT/2 | TT/1 | GG/3 |  |
|  |  | 460 | CG/2 | CC/4 |  |  |
| 25916 | AGCCTTGGGGACATCTTCTT | 259 | AT/2 | AA/1 | TT/3 |  |
|  | TGGACCCCTTAGACCTGTTG |  |  |  |  |  |
| 8539 | TGCTCATCCTTGGGCTCTAC | 1946 | AG/1 | AA/4 | GG/1 |  |
|  | TGCAGAGGATGGCTACAGTG | 2084 | CG/2 | CC/4 |  |  |
| 26657 | AAGCCCATTCTGATTGATGC | 337 | AG/1 | GG/5 |  |  |
|  | GGGTGCAACATTTACCCTTG | 490 | CC/6 |  |  |  |
| 2799 | GTTTCTGGCGCCGTAGATAG | 1584 | AT/2 | TT/2 | AA/1 | TT/1 |
|  | AACTGCCATGATCTCCCAAG |  |  |  |  |  |
| 23866 | AATCGGTCAGGACATTCAGC | 333 | CC/6 |  |  |  |
|  | GTGGGCAATAACGACGAGTT | 231 | AT/1 | AA/3 | TT/1 |  |
| 24919 | AGCTTAGGGGTGATGTGGTG | 497 | AA/6 |  |  |  |
|  | GAGCCAGTAACATGTCTTGCAT | |  |  |  |  |
| 13743 | GTTCCGTGGGGAGTTTGTTA | 960 | CT/4 | TT/1 | CC/1 |  |
|  | CACCCACAGACCTCCAATCT |  |  |  |  |  |
| 10466 | GGCAAGACTGCAAGTGGATT | 372 | AG/3 | GG/3 |  |  |
|  | AGAGACAGCACAGCCTGGAT | 108 | CT/1 | CC/5 |  |  |
| 12449 | CCGTAGAAACCGAAGACGAG | 524 | CG/4 | CC/1 | GG/1 |  |
|  | AGCCATTCTTGCTGCTTGTT |  |  |  |  |  |
| 4535 | GAAACATTTCCGCACCAAAT | 965 | CT/2 | TT/4 |  |  |
|  | CAGTGCTTGTGCAATCCAGT |  |  |  |  |  |
